# LIFETIME AND CURRENT CANNABIS USE BY OLDER WOMEN WITH HIV PARTICIPATING IN THE MACS/WIHS COMBINED COHORT STUDY

**DOI:** 10.1093/geroni/igae098.1243

**Published:** 2024-12-31

**Authors:** Julie Bobitt

**Affiliations:** University of Illinois Chicago, Chicago, Illinois, United States

## Abstract

The proportion of persons living with HIV who use cannabis is 2-3 times higher than the general population with past-year use rates ranging from 23%-56% in persons with HIV compared to 13% in persons without. A Women’s Interagency HIV Study (WIHS) found cannabis use by women to be 27%. We will present findings from interviews with 30 women aged 60+ enrolled in the MWCCS Cook County site. Guided by the socioecological framework, the interviews explore the multi-level influences on cannabis use decision-making and health outcomes. Our thematic analysis focuses on the following influences: 1) individual (attitudes, knowledge, health conditions); 2) Relationship (social connections/responsibilities); 3) Community (medical), and 4) Societal (legalization). We also explore cannabis use drivers on HIV-related pain relief, anxiety, and stress. As cannabis use among chronically ill persons continues to rise, it is important to understand the factors that influence use and health outcomes.

